# Inhibition of the Mitochondrial Permeability Transition for Cytoprotection: Direct *versus* Indirect Mechanisms

**DOI:** 10.1155/2012/213403

**Published:** 2012-05-22

**Authors:** Cécile Martel, Le Ha Huynh, Anne Garnier, Renée Ventura-Clapier, Catherine Brenner

**Affiliations:** LabEx LERMIT, INSERM U769, Faculté de Pharmacie, Université Paris-Sud, 5 Rue J.-B. Clément, 92290 Châtenay-Malabry, France

## Abstract

Mitochondria are fascinating organelles, which fulfill multiple cellular functions, as diverse as energy production, fatty acid **β** oxidation, reactive oxygen species (ROS) production and detoxification, and cell death regulation. The coordination of these functions relies on autonomous mitochondrial processes as well as on sustained cross-talk with other organelles and/or the cytosol. Therefore, this implies a tight regulation of mitochondrial functions to ensure cell homeostasis. In many diseases (e.g., cancer, cardiopathies, nonalcoholic fatty liver diseases, and neurodegenerative diseases), mitochondria can receive harmful signals, dysfunction and then, participate to pathogenesis. They can undergo either a decrease of their bioenergetic function or a process called mitochondrial permeability transition (MPT) that can coordinate cell death execution. Many studies present evidence that protection of mitochondria limits disease progression and severity. Here, we will review recent strategies to preserve mitochondrial functions via direct or indirect mechanisms of MPT inhibition. Thus, several mitochondrial proteins may be considered for cytoprotective-targeted therapies.

## 1. Introduction

Mitochondria are intracellular organelles, whose first discovered function is energy production by oxidative phosphorylation [1]. Depending on the mammalian tissue, mitochondria may have additional functions, such as *β* oxidation, heat production, reactive oxygen species (ROS) metabolism, and cell death coordination. However, since the emergence of the concept of mitochondrial control of cell death in the 95's (for recent reviews: [2, 3]), it became evident that mitochondria participate to various types of cell death, that are, apoptosis, necrosis, oncosis and mitotic catastrophy via mitochondrial membrane permeabilization (MMP), release of proapoptotic factors contained in the intermembrane space to the cytosol and possibly fission, even if mitochondrial fragmentation is not sufficient *per se *to induce cell death [4, 5]. Mitochondrial dysfunction has been associated with a series of human diseases such as cancer, cardiopathies, nonalcoholic fatty liver diseases, neurodegenerative diseases, and aging. When due to genetic dysfunction, the diseases have been systematically characterized in animal models [6, 7]. Thus, mitochondrial impairment can be linked either to the metabolic function of these organelles, their role in cell death, or both. In addition, in chronic pathologies, such as cardiac volume overload-induced hypertrophy [8], mitochondrial dysfunction precedes cell loss by apoptosis and necrosis, meaning that both dysfunctions can be separated chronologically during the progression of the disease. This is also observed in the pathogenesis of nonalcoholic steatohepatitis, whatever its initial cause, as extensively reviewed [9]. Hepatic mitochondrial dysfunction would lead to apoptosis or necrosis depending on the energy status of the cell [9]. 

Metabolic impairment manifests by decreased ATP synthesis capacity, enhanced ROS production due to electron leak from the respiratory chain, change in intracellular pH and frequently, by morphological alterations of mitochondrial network. For instance, heart failure which is defined as the inability of the heart to keep up with the demands and to provide adequate blood flow to other organs such as the brain, liver, and kidneys is accompanied by a decrease in energy production and energy transfer capacity [10]. This leads to a decrease in energy charge of the myocardium that has been described as a prognostic factor in dilated cardiomyopathies [11]. This metabolic impairment also affects the peripheric circulation and was shown to involve decreased mitochondrial biogenesis [10].

MMP corresponds to multiple events that irreversibly lead to cell death [2, 12]. These lethal events are nonexclusive, some of them can occur independently, whereas others are intimately linked. Thus, MMP refers to protein translocation from cytosol to outer membrane (OM), rupture of outer mitochondrial membrane, loss of inner membrane potential (ΔΨm), cristae remodeling and release of intermembrane space proteins such as cytochrome c or apoptosis-inducing factor (AIF). For instance, upon various stress, Bax or tBid, which reside in the cytosol, can translocate to mitochondrial membranes, oligomerize with mitochondrial proteins to form large channels allowing cytochrome c release and activation of the intrinsic apoptosis signaling cascade (for review: [2]).

In many pathophysiological models, but not all, MMP also involves the so-called opening of the permeability transition pore complex (PTPC), which mediates a nonselective permeabilization of the IM and OM to molecules of molecular mass (MM) under 1.5 kDa (see below for more details) [2, 12]. Thus, in chemotherapy-treated tumor cell lines and ischemic neuronal cells, Bax can interact with the adenine nucleotide translocase (ANT) and/or the voltage-dependent anion channel (VDAC) to promote MMP and cell death.

Here, we will review direct and indirect mechanisms or means to protect mitochondrial functions via a closure of PTPC and a prevention of mitochondrial permeability transition (MPT). The discussion of MPT regulatory mechanisms will be based on selected articles focusing on heart diseases and cancer.

## 2. Mitochondrial Membrane Permeability and PTPC

**Figure 1:**
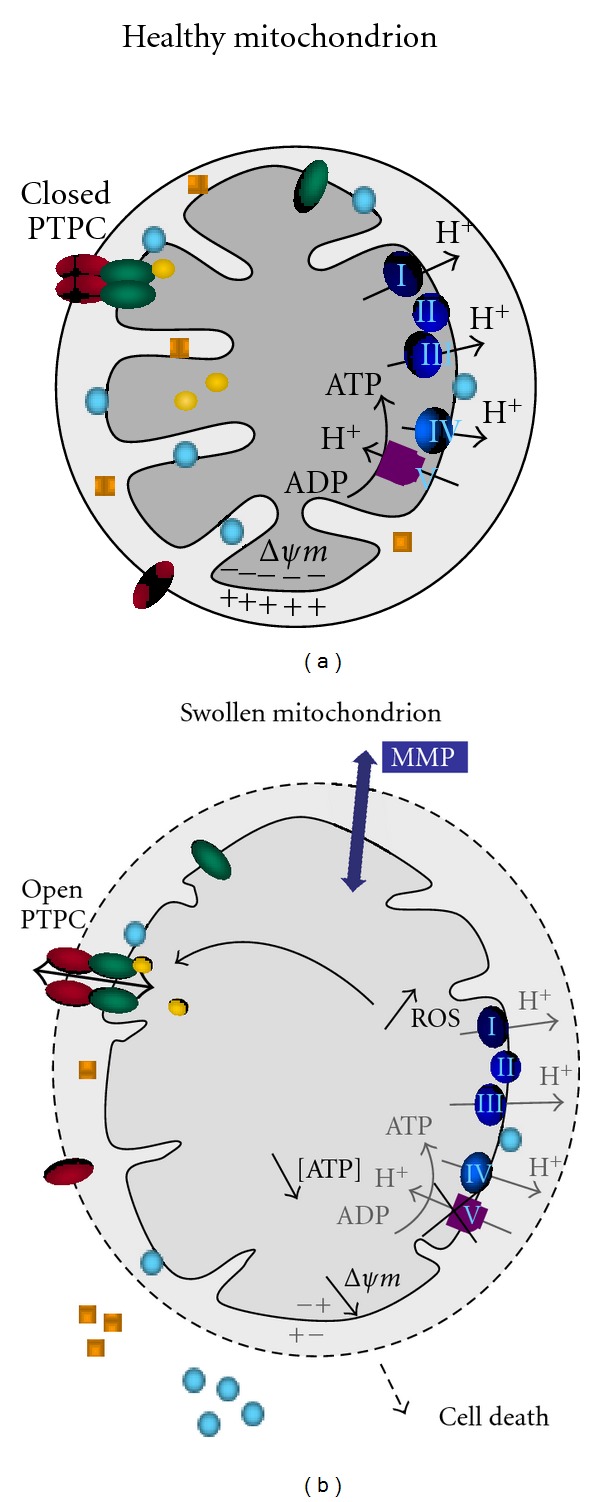
Scheme of mitochondrial alterations following mitochondrial membrane permeabilization (MMP). In this model, in response to the opening of the permeability transition pore (PTPC; green and red ellipses, corresponding to ANT and VDAC resp.), swollen mitochondria exhibit an increase in volume, a more translucide matrix with less cristae and a permeabilized outer membrane. Cytochrome c and apoptosis-inducing factor (AIF) (blue circles and yellow squares), normally confined into the intermembrane space, are released trough ruptures in the outer membrane. The transmembrane inner potential (ΔΨm) is dissipated in response to the arrest of the function of the respiratory complexes (I to V), which contributes to an inhibition of ATP biosynthesis. Altogether, these alterations are lethal, irreversible and lead to cell death.

Mitochondrial membrane permeability is strictly controlled in unicellular and multicellular organisms harboring these organelles. The OM is believed to be freely permeable to ions and metabolites via entry *through* protein channels (e.g., voltage-dependent anion channel, VDAC), whereas the inner membrane (IM) is considered as impermeable. Thus, the entry and exit of ions or metabolites trough the IM are mediated by integral proteins such as the members of the mitochondrial carrier family [13]. The prototypic protein of this family is ANT or ADP/ATP carrier, which mediates the stoichiometric exchange of ADP and ATP between the matrix and the intermembrane space [14]. Moreover, osmotic movements of water accompany solutes transport from cytosol to matrix, but the molecular basis of these transports is still largely unknown [15]. When excessive stimulation by endogenous signals (excessive ROS, calcium (Ca^2+^) overload, protease activation, lipid accumulation etc) or activation of harmful signaling pathways (e.g., kinases/phosphatases, proteases, Bax/-Bid-mediated pathways etc.) occur, mitochondria undergo the MPT, a phenomenon that consists in a sudden increase in IM permeability to small molecules. MPT is a phenomenon first studied in isolated beef heart mitochondria in response to Ca^2+^ overload [16]. Thus, the response of isolated mitochondria to doses of Ca^2+^ is a nonspecific increase of the permeability of the IM, resulting in entry of water and solutes, loss of ΔΨm, matrix swelling, and simultaneous uncoupling of oxidative phosphorylation (Figure 1). Of note, the doses of Ca^2+^ depend largely on the tissue origin of mitochondria and the amount of Ca^2+^ present in the buffers. Ultimately, MPT is accompanied by matrix swelling and OM ruptures as shown by transmission electron microscopy [17–19]. This phenomenon can be blocked by Ca^2+^ chelation, ATP, Mg^2+^, and cyclosporin A (CsA) *in vitro *as well as *in vivo *[20–22]. 

MPT can be followed experimentally by the loss of absorbance of a suspension of isolated mitochondria by spectrophotometry and by the loss of ΔΨm using suitable fluorescent probes (e.g. tetramethylrhodamine methyl ester (TMRM), rhodamine 123 (Rhod123), 5, 5′, 6, 6′- tetrachloro-1, 1′, 3, 3′-tetraethylbenzimidazol-carbocyanine iodide (JC-1)) [19, 23]. The main interests of the use of isolated organelles are to monitor mitochondrial responses that are directly induced by compounds independently of other cellular compartments and the possibility to automate the measure in the perspective of pharmacological studies [19, 23].

One major pitfall is that, whereas isolation procedures are believed to be nondestructive for liver and cell lines mitochondria [24, 25], mitochondrial responses of isolated mitochondria from skeletal muscle and heart that may rely on the cell architecture are (obviously) lost [26].

Another pitfall is the cross-contamination of the mitochondrial fraction with other cellular compartments and purity of preparations must be checked carefully. MPT can also be measured by imaging with the fluorescent probe calcein in the presence of cobalt in living cell as various as hepatocytes, astrocytes and cardiomyocytes [27–31]. The principle is that calcein (molecular weight, 620 Da) can diffuse into the whole cell, whereas cobalt, a fluorescence quencher, cannot enter into the mitochondrial matrix and diffuses into the rest of the cell. Thus, in physiological conditions mitochondria appear fluorescent and following MPT, the quenching of calcein by cobalt triggers a decrease in fluorescence. For instance, HeLa cells treated by thapsigargin, a SERCA pump inhibitor or A23187, a Ca^2+^ ionophore [32], undergo MPT as shown by a significant decrease in calcein fluorescence due to IM permeabilization and cobalt quenching [30]. MPT has also been monitored in whole heart by 2-deoxy[^3^H]glucose entrapment technique [33].

Of note, the full demonstration that the process is mediated by PTPC opening requires its inhibition by pretreatment of cells or isolated mitochondria by CsA, the well-known cyclophilin D (CypD) ligand [20, 34]. Moreover, silencing of CypD by siRNA to prevent the induction of MPT is becoming mandatory *in cellulo*, since the genetic demonstration that CypD is critical for MPT and cell death [35].

## 3. Direct Mechanisms of MPT Inhibition

PTPC is defined as a voltage-dependent polyprotein complex, which in certain conditions might form a nonselective channel at contact sites between both mitochondrial membranes [36, 37]. By definition, mitochondria contain all the proteins necessary for MPT induction and then MPT does not necessitate any neosynthesis. Since the initial PTPC identification by electrophysiology, the molecular identity of this pore and its regulators is still controversial [38–40]. ANT, VDAC and CypD, the three former PTPC candidates, have their own functions, irrespective of their association within PTPC or other putative polyprotein complexes such as the ATP synthasome [41] and Bcl-2 family member oligomers [3, 12]. Thus, some of the unknown members of PTPC may have their own role in metabolism (e.g., kinase, peptidyl-prolyl isomerase, deshydrogenase), transport (e.g., mitochondrial carrier, channel) or structure (e.g., dynamic machinery, cytoskeleton, AKAP proteins). This means that lethal MPT needs a stimulation to occur and in pathophysiological conditions, this is mainly achieved by Ca^2+^ and ROS. Whatever its composition, the PTPC is a widespread phenomenon occurring in many diseases. Although it has been the subject of intense research and therapeutic developments in cancer with the search for PTPC inducers [42], it has emerged only recently as a promising target for cytoprotection in various diseases such as neurodegenerative, cardiovascular and metabolic diseases. PTPC can be modulated directly by a large panel of pharmacological agents, by post-translational modifications and by cooperation with other proteins that may have a major impact on the cell life as discussed below. 

### 3.1. Pharmacological Inhibition of PTPC

Using isolated mitochondria from various sources, an impressive body of literature reports that many molecules or compounds modulate the PTPC in response to Ca^2+^, ROS, or a disease. Thus, some compounds can activate MPT, whereas a more limited number of them can prevent the opening of PTPC. Some compounds have known mitochondrial targets such as ANT, VDAC, CypD, and translocator protein 18 kDa (Table 1). To summarize, the most investigated inhibitor of PTPC is CsA, which modulates CypD, as discussed below.

One example with future therapeutic applications is cardiac ischaemia/reperfusion injury. During an acute myocardial infarction (AMI), tissue injury occurring after reperfusion represents a significant amount of the whole, irreversible damage. Ischaemia and reperfusion cause a wide array of functional and structural alterations of mitochondria. Some of these responses are directly under the control of the highly conserved transcriptional complex HIF-1 and result from a modulation in expression of genes involved in glycolysis, glucose metabolism, mitochondrial function, cell survival, apoptosis, and resistance to oxidative stress [43]. 

PTPC opening plays a crucial role in this specific component of myocardial infarction. Strong support for this concept has recently been provided by the reduced infarct size observed in mice lacking CypD [35]. Thus targeting PTPC appears a relevant strategy to reduce ischaemic damages at reperfusion. A large body of evidence has shown that it is possible to reduce infarct size and to protect the heart after an infarct by a postconditioning or pharmacological strategy. Brief episodes of myocardial ischemia-reperfusion employed during reperfusion after a prolonged ischaemic insult may attenuate the total ischaemia-reperfusion injury. Recently, CsA has been shown to dramatically reduce infarct size in many animal species and in human. Recent proof-of-concept clinical trials support the idea that targeting MPT by either coronary intervention postconditioning or CsA can reduce infarct size and improve the recovery of contractile function after reperfusion [21, 73]. Such a strategy was also applied to ischaemia-reperfusion damages in other tissues and cells like vascular endothelial cells [74], hepatocytes [75, 76], and neurons [77–79]. Moreover, some attempts to target CsA to the mitochondrial compartment by conjugation to the lipophilic triphenylphosphonium cation proved to be promising in cytoprotection from glucose and oxygen deprivation in neurons [80], in cardiomyocytes [81], and in other various organs [82]. 

### 3.2. Role of Mitochondrial Kinases to Prevent PTPC Opening

Several protective signal pathways involving multiple kinases have been shown to converge on mitochondria and the PTPC [83] to promote cell survival (Table 2). For instance, in cardiomyocytes, pools of kinases such as Akt, protein kinase C-*ε* (PKC*ε*), extracellular-regulated kinases (ERK), glycogen synthase kinase-3 beta (GSK-3*β*), and hexokinases (HK) I and II, are localized in or on mitochondria in addition to the cytosol. These mitochondria-associated protein kinases may integrate cytosolic stimuli and in turn, enhance tolerance of myocytes to injury. 

Moreover, systematic proteomic approaches revealed that some of these kinases might form hetero-oligomers with putative components of the PTPC. Briefly, GSK-3*β* and HKs are directly responsible for inhibition of opening of the PTPC and, thus, for myocyte protection from necrosis [96]. As a result, postconditioning, which leads to GSK-3*β* inhibition, allows the myocardial salvage from reperfusion injury by modulating MPT [86]. In the context of anticancer chemotherapy, *β*-adrenergic receptors (*β*-ARs) modulate anthracycline response through crosstalk with multiple signaling pathways. *β*2-ARs are cardioprotective during exposure to oxidative stress induced by doxorubicin (DOX). DOX cardiotoxicity is mediated in part through a Ca^2+^-dependent triggering of MPT as clearly shown by a 41% reduction of DOX-induced mortality by CsA [97]. *β*2-ARs activate prosurvival kinases and attenuate mitochondrial dysfunction caused by oxidative stress. Accordingly, the invalidation of *β*2-ARs enhances cardiotoxicity via negative regulation of survival kinases and enhancement of intracellular Ca^2+^, thus predisposing the mitochondria to opening of the PTPC [97].

Moreover, in cancer cell lines, activation of mitochondrial ERK protects cancer cells from death through inhibition of the MPT [88]. ERK inhibition enhanced GSK-3*β*-dependent phosphorylation of the pore regulator CypD, whereas GSK-3*β* inhibition protected from PTPC opening.

By different molecular mechanisms, some kinases such as creatine kinase (CK) and HK have also cytoprotective effects and prevent PTPC opening. Depending on the tissue, which supports their expression, these kinases may be cytoprotective via a role in energy transfer to metabolites such as creatine and glucose (Table 2).

### 3.3. Stabilization of Mitochondrial Membrane Permeability by Bcl-2 Family Members

The Bcl-2 family is composed of more than 25 proteins implicated in the control of life-or-death decision [98]. This protein family has been particularly studied in cancer, which led to the classification of Bcl-2 and Bax as oncogenes and tumor-suppressors, respectively. Some members (e.g., Bax/Bad proteins, BH3-only proteins) favor apoptosis, whereas other members, such as Bcl-2 and Bcl-XL, prevent apoptosis. Moreover, it has been shown that the effects of Bcl-2 family proteins on mitochondria in cancer cells are linked to clinical responses to chemotherapy [99]. 

The cytoprotective mechanisms of Bcl-2 family members are multiple and include direct mitochondrial effects [3, 12]. Thus, Bcl-2 contributes to the stabilization of the mitochondrial membrane permeability, inhibition of ΔΨm loss and cytochrome *c* release and, at least in tumor cells, to the stimulation of oxidative phosphorylation [100–103]. Thus, direct protein-protein interactions between Bcl-2 family members, but also with several constitutive mitochondrial proteins such as ANT (IM), VDAC (OM), or FoF1-ATP synthase (IM) have been evidenced [104]. Bcl-2 cooperates directly with ANT, to prevent PTPC opening and to inhibit cell death [101, 105, 106]. In addition, Bcl-2 blocks the Ca^2+^-induced channel function of ANT and favors ADP/ATP translocase function, which positively impacts the intracellular levels of ATP [107]. Similarly, Bcl-2 and Bcl-XL directly interact with VDAC modulating its channel function [108]. Bcl-XL would bind also to the mitochondrial F0F1-ATP synthase and regulate metabolic efficiency in neurons [104]. Accordingly, recombinant proteins of some members of the Bcl-2 family directly modulate PTPC in isolated mitochondria from various sources, that is, liver and cancer cells [25, 109]. Some specific regions of Bcl-2 (e.g., BH3, BH4 domains) are responsible for these effects and as expected, peptides corresponding to these regions proved to modulate apoptosis *in cellulo *or in isolated mitochondria, again indicating a direct targeting of mitochondria [25]. Finally, *in vivo*, the BH4 domain of Bcl-XL exerts antiapoptotic effects and attenuates ischaemia/reperfusion injury through anti-apoptotic mechanism in rat hearts [110, 111].

## 4. Indirect Mechanisms, Which Lead to an Increased Resistance of PTPC Opening

By definition, several indirect mechanisms may lead to blockade of PTPC opening via modulation of ΔΨm, mitochondrial mass regulation, redox state, fusion/fission processes and calcium retention capacity. This may be due to modification in protein expression, in posttranslational modifications and in their interactome, which consequently affect signaling pathways. Below, we will analyze three indirect mechanisms of PTPC protection that have recently been elucidated. 

### 4.1. Anti-Oxidant Protection

Mitochondria are major sites of ROS production, which may contribute to the development of various diseases including cardiovascular diseases and aging. Several studies have thus described the effects of antioxidant administration in the context of cardiac and liver pathologies in mice [112]. It is widely admitted that natural antioxidants such as resveratrol and curcumin have beneficial effects against ischaemia/reperfusion damages to mitochondria and cells in rat liver or heart [113, 114]. These effects are complex since resveratrol has been proposed to have multiple intracellular targets such as AMPK, SIRT1 and Nrf2, which can influence the transcriptome to increase the anti-oxidant defense (e.g., catalase, GPx, and GCLC), and other genes such eNOS and PGC1*α*, which favor an increase in mitochondrial mass and bioenergetics and decrease in apoptosis and inflammation (for review: [115]).

Resveratrol treatment exerts beneficial protective effects on survival, endothelium-dependent relaxation, and cardiac contractility and mitochondrial function, suggesting that resveratrol or metabolic activators could be a relevant therapy in hypertension-induced heart failure [116]. Similarly, in the heart, curcumin another polyphenol with antioxidant properties showed cardioprotective effects in catecholamine induced cardiotoxicity through prevention of mitochondrial damage, PTPC opening [117], and ventricular dysfunction [118] and in protecting rat myocardium against ischaemic insult by decreasing oxidative stress [119].

**Figure 2:**
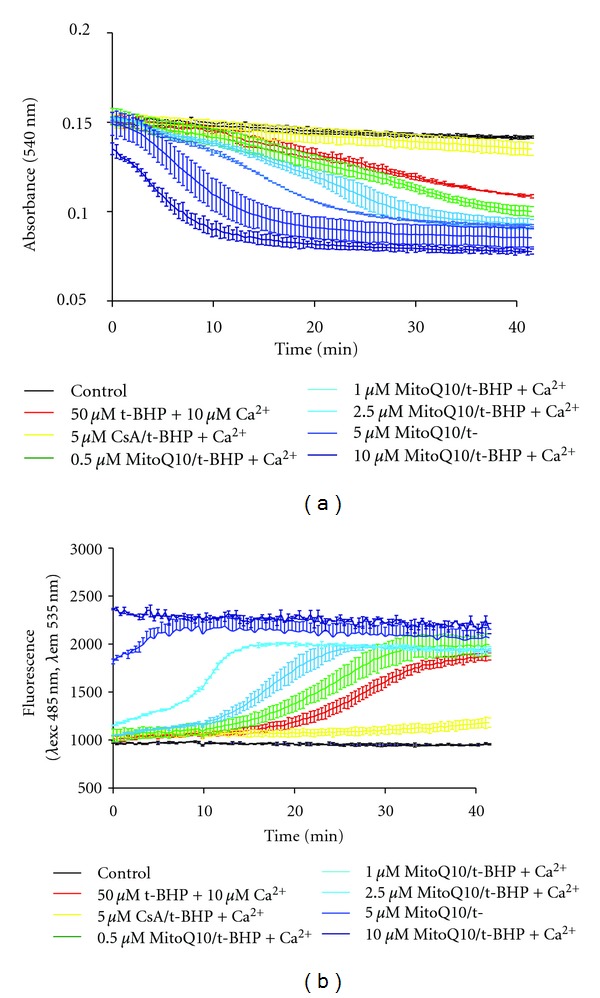
MitoQ10 stimulates MPT in isolated cardiac mitochondria. (a) MitoQ10 increases the mitochondrial swelling induced by an oxidant stress. Mitochondria (25 *μ*g of proteins) have been pretreated by the indicated doses of MitoQ10 and treated by 50 *μ*M t- BHP + 10 *μ*M Ca^2+^. Absorbance at 540 nm has been registered for 60 min at 37°C. (b) MitoQ10 increases the mitochondrial depolarization induced by an oxidant stress. Mitochondria (25 *μ*g of proteins) have been loaded with 2 *μ*M Rhodamine 123, pretreated by the indicated doses of MitoQ10, and treated by 50 *μ*M t-BHP + 10 *μ*M Ca^2+^. Fluorescence has been registered for 60 min at 37°C.

Another promising example of compound is MitoQ10, an ubiquinone derivative, which is a mitochondria-targeted antioxidant [120]. It has proven to be useful for protecting endothelial function and attenuating cardiac hypertrophy in stroke-prone hypertensive rats [121]. Moreover, MitoQ10 potently inhibits cocain-induced cardiac damage via a restoration of oxygen consumption and a stabilization of ROS levels, specifically in interfibrillar mitochondria [122]. However, when used on isolated cardiac mitochondria, MitoQ10 can be enhanced in a dose-dependent-manner MPT in the presence of the prooxidant tert-butyl hydoperoxide (t-BHP) and suboptimal doses of Ca^2+^ (Figure 2), although it acts as an antioxidant on rat liver mitochondria [123]. This underscores the duality of anti- and prooxidant compounds, whose effects can depend either on the dose, the redox state of the cell, the tissue, and/or the mode of administration. This probably explains, at least in part, the failure of anti-oxidants to protect efficiently the heart function in clinical trials, as recently reviewed in [124].

### 4.2. Estrogens Protection

Sex and gender influence the onset and the progression of many human diseases, notably age-related diseases. Thus, estrogens, mainly 17*β*-estradiol, may have pleiotropic effects depending on the tissue [125]. For instance, certain cardiovascular diseases, such as myocardial hypertrophy and heart failure, differ clearly in their clinical manifestation and prognosis between women and men [126]. As a consequence, hormonal mechanisms underlying sex and gender differences are currently under intense investigation.

Animal and cellular models have been particularly instrumental to better understand estrogen protection at the level of mitochondria [127]. For instance, in cerebral circulation, estrogens mediate an enhancement of vasodilator capacity, suppression of vascular inflammation and increase of mitochondrial efficiency [125, 128, 129]. This effect is, at least in part, due to an increase in mitochondrial biogenesis via gene expression modulation [129] and a decrease in superoxide production [125]. Accordingly, chronic estrogen treatment increases mitochondrial capacity for oxidative phosphorylation while decreasing production of ROS. In breast and lung cancer cells, long-term estradiol treatment activates transcription of NRF-1 and increases mitochondrial biogenesis [130].

Moreover, mitochondrial effects on PTPC might be mediated indirectly by estrogen receptors, *α* and *β*, present in nucleus, plasma membrane, endoplasmic reticulum and even mitochondria [131]. In the context of ischaemia-reperfusion injury, it is widely admitted that estrogens protects from myocardial damage via an inhibition of PTPC function. Notably, estradiol may activate the signaling cascade which involves Akt, NO synthase, guanylyl cyclase and protein kinase G, which results in blockade of MPT-induced release of cytochrome c from mitochondria, respiratory inhibition and caspase activation [131]. As a result, estrogens effects are multifactorial, mostly indirect. Even if some estrogen-like molecules can be effective on isolated mitochondria, a precise target of estrogen within PTPC is still unknown [132]. Thus, intriguingly, estrogens may prevent Ca^2+^-induced cytochrome *c* release in isolated heart mitochondria, but not mitochondrial swelling [133].

Estrogens also protect from chemotherapy-induced cardiomyopathy in ovariectomized rats. Again, effects on the anti-oxidant cellular defenses have been proposed as one of the target mechanism of estrogen [134].

### 4.3. Exercise Protection

Exercise training has proven to be beneficial in chronic diseases including heart failure, obesity, diabetes or metabolic syndrome. Because endurance training improves symptoms and quality of life and decreases mortality rate and hospitalization, it is increasingly recognized as a beneficial practice for these patients. Adaptation to endurance training mainly involves energetic remodeling in skeletal and cardiac muscles [135]. The mechanisms involved in the beneficial effects of exercise training are far from being understood. Skeletal muscles adapt to repeated prolonged exercise by marked quantitative and qualitative changes in mitochondria. Endurance training promotes an increase in mitochondrial volume density and mitochondrial proteins by activating mitochondrial biogenesis [136]. Exercise training decreases apoptotic processes, and protects mitochondrial function from oxidative stress and other cardiac insults [137, 138]. Exercise training results in a reduced sensitivity to PTPC opening in heart mitochondria and confers mitochondrial protection. Moreover, even acute exercise protects against cardiac mitochondrial dysfunction, preserving mitochondrial phosphorylation capacity and attenuating DOX-induced decreased tolerance to PTPC opening [139]. Proposed mechanisms to explain the cardioprotective effects of exercise are mediated, at least partially, by redox changes and include the induction of myocardial heat shock proteins, improved cardiac antioxidant capacity and/or elevation of other cardioprotective molecules [137].

## 5. Conclusion and Open Questions

In the last decade, direct and indirect approaches to protect mitochondrial functions via PTPC modulation have been explored. However, it is still too early to decipher the most efficient strategies in term of cytoprotection. Nevertheless, recent studies and research advances have propelled mitochondria on the scene front of new therapeutic strategies. However, a contradiction emerges between the need to kill tumor cells in cancer therapy and to protect other cells from injuries. Even more worrying is the fact that many anticancer therapies have mitochondrial toxicity that becomes dramatic when highly oxidative nondividing cells like cardiomyocytes are concerned. Indeed, mitochondria are the main target when cardiotoxicity of anticancer drugs is concerned [44, 140]. Thus one challenging issue of cytoprotection directed to mitochondria would be to uncover new molecules or treatments that would selectively target cancer cells without affecting cardiac mitochondria. This should stimulate new studies devoted to increase our basic knowledge of the mechanisms and the tissue specificity of PTPC opening and mitochondrial function. At the same time, this will open the possibility to search for new drugs with tissue-specific effects on mitochondria. Finally, another challenge that basic and clinical research will face in the future is the notion of sex and gender influence that might be decisive for the treatment of many severe diseases.

## Figures and Tables

**Table 1:** List of mitochondrial permeability transition (MPT) inhibitors. CypD, Cyclophilin D; VDAC, voltage-dependent anion channel; ANT, adenine nucleotide translocase; UQ, ubiquinone.

MPT inhibitor	Target	Model	References
Cyclosporin A	CypD binding with ANT	*In vivo*, isolated mitochondria, cells	[44, 45]
Sanglifehrin A	CypD	*In vivo*, isolated mitochondria, cells	[46, 47]
Bongkrekic acid	ANT	Isolated mitochondria, cells	[48–50]
ADP/ATP	ANT	Isolated mitochondria, *in vitro *	[51, 52]
NADH/NAD+	VDAC	Isolated mitochondria,* in vitro *	[53–55]
DIDS	VDAC	Isolated mitochondria, *in vitro*, cells	[52–58]
glutamate	VDAC	Isolated mitochondria, *in vitro *	[59–61]
Ro 68–3400	ANT or PiC, not VDAC1	Isolated mitochondria, *in vitro *	[61–63]
UQ(0)	ANT or PiC	Isolated mitochondria, *in vitro *	[64, 65]
S15176	unknown, in IM	*In vivo*, isolated mitochondria	[66, 67]
Sildenafil	unknown	*In vivo*, isolated mitochondria	[68]
Debio-025	CypD	*In vivo, *isolated mitochondria	[69]
TRO19622	VDAC, translocator protein 18 kDa	*In vivo, *isolated mitochondria	[70]
Carbon monoxide	ANT, unknown	Isolated mitochondria, cells	[71]
Antamanide	CypD	Isolated mitochondria, cells	[72]

**Table 2:** List of kinases contributing to a closure of PTP via phosphorylation mechanisms or protein-protein interaction. HK, hexokinase, CK, creatine kinase, PKG, protein kinase G, PKA, protein kinase A, PKC, protein kinase C, ERK, extracellular signal-regulated kinase, GSK3, glucose-regulated kinase 3, PI3K, phosphoinositol3 kinase, and Akt/PKB, protein kinase B.

Kinase	Effect	Target/pathway	Model	References
Akt/PKB, PI3K	Indirect	GSK3 via PI3K or eNos/PKG pathways	Cells	[84, 85]
GSK3	Direct	VDAC, ANT, CypD	Isolated mitochondria, cells,* in vivo*, *in vitro *	[8, 37, 86, 87]
ERK	Indiret	GSK3 via PI3K pathway	Cells	[85, 88]
PKA	Direct	VDAC	Isolated mitochondria	[89]
PKC epsilon	Direct	VDAC	Isolated mitochondria, *in vivo *	[90]
PKG	Direct	Unknown	Isolated mitochondria, *in vivo *	[91, 92]
CK	Local regulation of ATP/creatine pools	Energetic metabolism	CK-expressing tissues	[93, 94]
HK	Local regulation of glucose/ATP pools	Energetic metabolism	Isolated mitochondria, cells, *in vitro *	[57, 74, 87, 95]

## Abbreviations

ANT: Adenine nucleotide translocase
*β*-Ars: *β*-adrenergic receptors
Ca^2+^: Calcium
CK: Creatine kinase
CsA: Cyclosporin A
CypD: Cyclophilin D
ΔΨm: Mitochondrial inner membrane potential
DOX: Doxorubicin
ERK: Extracellular-signal-regulated kinase
GSK-3*β*: Glycogen synthase kinase-3 beta
HK: Hexokinase
IM: Inner membrane
JC-1: 5, 5′, 6, 6′ -tetrachloro-1, 1′, 3, 3′ -tetraethylbenzimidazol-carbocyanine iodine
MMP: Mitochondrial membrane permeabilization
MPT: Mitochondrial permeability transition
OM: Outer membrane
PTPC: permeability transition pore complex
Rhod123: Rhodamine 123
ROS: Reactive oxygen species
TMRM: Tetramethylrhodamine methyl ester
VDAC: Voltage-dependent anion channel.
